# Standardized intensive care unit management in an anhepatic pig model: new standards for analyzing liver support systems

**DOI:** 10.1186/cc9196

**Published:** 2010-07-22

**Authors:** Christian Thiel, Karolin Thiel, Alexander Etspueler, Thomas Schenk, Matthias H Morgalla, Alfred Koenigsrainer, Martin Schenk

**Affiliations:** 1 Department of General, Visceral and Transplant Surgery, Tübingen University Hospital, Hoppe-Seyler-Str. 3, Tübingen, D-72076, Germany; 2 Department of Anaesthesiology, Tübingen University Hospital, Hoppe-Seyler-Str. 3, Tübingen, D-72076, Germany; 3 Department of Neurosurgery, Tübingen University Hospital, Hoppe-Seyler-Str. 3, Tübingen, D-72076, Germany

## Abstract

**Introduction:**

Several anhepatic pig models were developed in the past. Most models suffer from short anhepatic survival times due to insufficient postoperative intensive care unit (ICU) management. The aim of this study was to analyze anhepatic survival time under standardized intensive care therapy in a pig model.

**Methods:**

Eight pigs underwent total hepatectomy after Y-graft interposition between the infrahepatic vena cava and the portal vein to the suprahepatic vena cava. An intracranial probe was inserted for intracranial pressure (ICP) monitoring. Animals received pressure-controlled ventilation under deep narcosis. Vital parameters were continuously recorded. Urinary output, blood gas analysis, haemoglobin, hematocrit, serum electrolytes, lactate, and glucose were monitored hourly, and creatinine, prothrombin time, international normalised ratio, and serum albumin were monitored every 8 hours. Sodium chloride solution 0.9%, hydroxyethyl starch 6%, fresh frozen plasma, and erythrocyte units were used for volume substitution, and norepinephrine was used to prevent severe hypotension. Serum electrolytes and acid-base balance were corrected as required. Antibiotic prophylaxis with ceftriaxon was given daily, as well as furosemide, to maintain diuresis.

**Results:**

Postoperative survival was 100% after 24 hours, with a maximum survival of 73 (mean, 58 ± 4) hours. Haemodynamic parameters such as heart rate, mean arterial pressure, and pulse oximetry remained stable during surgical procedures and following anhepatic status due to ICU therapy until escalating at time of death. Deteriorating pulmonary function could be stabilized by increasing oxygen concentration, positive end-expiratory pressure, and maximal airway pressure. Furosemide was used to maintain diuresis until renal failure occurred. ICP started at 15-17 mmHg and increased continuously up to levels of 41-43 mmHg at time of death. All animals died as a result of multiple-organ failure.

**Conclusions:**

Using standardized intensive care management after total hepatectomy, we were able to prolong anhepatic survival over 58 hours without the use of liver support systems. The survival benefit of liver support systems in previous animal studies should be reevaluated against our model.

## Introduction

Several models of acute hepatic failure have been investigated in animal studies [1-3] to analyze bioartificial [4-6] or artificial [7] liver support technologies as treatment options to bridge until transplantation or to support liver regeneration. In these models, hepatic failure was achieved by intoxication with galactosamine or acetaminophen [8-10], hepatic ischemia by portocaval shunting following transient clamping of the hepatic artery [11,12], and extended resection or even total hepatectomy [13-21]. Unfortunately, all aforementioned models involve various limitations, thus affecting morbidity in the assessment of a given intervention.

Advantages of the anhepatic model [22] are its reproducibility and its potential to assess the efficiency of artificial or bioartificial liver support systems in vivo in the absence of toxic products leaking out of or produced by the native liver. From the surgical point of view, the operation is technically complex but well reproducible. Our newly developed technique [23] allows total hepatectomy in haemodynamically stable animals due to lateral clamping of large blood vessels.

A review of the management of experimental anhepatic coma in various pig models showed several treatment strategies. They vary completely from almost no postoperative treatment at all [24] to mechanical ventilation and supportive therapy with colloid fluid resuscitation and catecholamines [19]. Considering this fact, it is astonishing that none of the previous anhepatic models established any standardized intensive care therapy or used supportive therapy, which has been established in humans for the past decade [25]. Furthermore, survival times between studies showed major variations from 10 to 45 hr. These survival rates may be judged as a control group because it is ethically not justifiable to kill animals to verify insufficient therapy.

Postoperative critical care medicine management by itself has an important impact on anhepatic survival. Moreover, well-intended established procedures such as fluid resuscitation may impair coagulation homeostasis, thus possibly complicating outcome. Therefore, it is important to use a reproducible animal model as a tool for screening a given intervention with high validity to improve hepatic failure outcome. The aim of our study was the establishment of a highly reproducible anhepatic pig model with standardized postoperative intensive care management, which offers long-term survival to evaluate new treatment strategies such as artificial or bioartificial liver support systems.

## Materials and methods

### Experimental design

After approval by the institutional review board for animal experiments, eight female German Landrace pigs weighing between 32 and 46 (mean, 37.4) kg underwent total hepatectomy. All experiments were performed according to the international principles governing research on animals and under the supervision of a veterinarian, who set the guidelines for minimizing the pigs' suffering.

### Anaesthesia

Intramuscular premedication was administered using atropine 0.1% (0.05 mg/kg), ketamine (7 mg/kg), azaperone (10 mg/kg), and diazepam (1 mg/kg). Adequate temperature (approximately 38.5°C) was maintained with a warming blanket. Two 18-gauge venous catheters (Vasofix; Braun Melsungen, Germany) were inserted into auricular veins for volume substitution preventing hypovolemia, and a 20-gauge central venous catheter (Cavafix; Braun Melsungen, Germany) was placed through one 18-gauge catheter for intravenous anaesthesia during the surgical procedure. A stomach tube (Argyle; Tyco Healthcare, Tullamore, Ireland) was placed for intestinal drainage. After oral intubation with a cuffed endotracheal tube (Lo-Contour Magill; Mallinckrodt Medical, Athlone, Ireland), the pigs were ventilated with pressure-controlled ventilation to deliver a tidal volume of 6-10 ml/kg with a respiratory rate of 8-12 breaths per minute (Galileo Gold; Hamilton Medical, Rhaezuens, Switzerland). Arterial blood gas analysis (ABL 625; Radiometer, Copenhagen, Denmark) was performed hourly, and ventilation was adjusted accordingly. Continuous infusion of ketamine (15 mg/kg/h), fentanyl (0.02 mg/kg/h), and midazolam (0.9 mg/kg/h) was administered to maintain anaesthesia during the study. Character of respiration, heart rate, eye movement, and pain stimulus was used to confirm depth of anaesthesia; if any of these parameters indicated a lessening of anaesthesia, infusion rates of anaesthetic agents were increased.

### Surgical procedure

In brief, animals were kept under standard laboratory conditions and fasted for 24 hr before surgery. They received an antibiotic prophylaxis of 2 g ceftriaxon (Rocephin; Hoffmann-La Roche, Basel, Switzerland). The superior vena cava through the jugular veins and the internal carotid artery were instrumented to measure arterial (Leadercath; Vygon, Écouen, France) and central venous pressure (Multi-Lumen Central Venous Catheter; Arrow International, Reading, PA, USA). Following parietofrontal cranial trepanation, a probe was inserted into the frontal brain parenchyma to measure intracranial pressure and brain temperature (Camino MPM-1 monitor; Integra Neurosciences, Plainsboro, NJ, USA). The abdominal cavity was entered through a midline abdominal incision, and a urinary catheter (Gentle-Flo; Tyco Healthcare, Tullamore, Ireland) was placed. The portal vein and the infrahepatic vena cava were mobilized and prepared for anastomosis with the Y-graft vascular prosthesis (Uni-Graft K DV; ITV, Denkendorf, Germany). The diaphragm was opened on the left side for the suprahepatic anastomosis. Performing an end-to-side anastomosis between the Y-graft and first the infrahepatic vena cava, then the portal vein, and finally the suprahepatic vena cava, lateral clamping was applied consecutively to only one third of the vessels. End-to-side anastomosis allowed partial clamping, thus preventing serious congestion of the intestine and a decline in systemic blood pressure. The supra- and infrahepatic vena cava and the portal vein were clamped totally and ligated, and blood flow was released through the bypass. Afterward, the hepatoduodenal ligament was ligated, and the liver was removed *en bloc*, including the retrohepatic vena cava. After achieving haemostasis, the abdominal wall was closed with a running suture. During surgery, sodium chloride solution 0.9% and hydroxyethyl starch 6% (Voluven HES 130/0.4; Fresenius, Bad Homburg, Germany) were infused, adjusted for mean arterial and central venous pressure. Blood loss caused by the blood volume remaining in the liver ranged from 300 to 700 ml and was substituted with donor erythrocytes and fresh frozen plasma units. Furosemide (1 mg/kg) was given to obtain high urine output during the surgical procedure. Two hours after surgery, the pigs were transferred to the animal critical care unit.

### Preparation of donor fresh-frozen plasma units and erythrocyte units

All donor pigs were tested for A-O blood group antigens to avoid A-O incompatible transfusion reactions. Blood was collected in standard blood bag systems (500 ml, Compoflex; Fresenius HemoCare, Bad Homburg, Germany) and centrifuged at 2500 *g *for 20 min (Heraeus Cryofuge 5500i; Thermo Electron Corporation, Langenselbold, Germany). Plasma fraction was pressed into separate bags and shock-frozen at -80°C. Erythrocytes were conserved with 100 ml SAG-M and stored at 4°C for a maximum of 7 days. Immediately before transfusion, a cross-match test was done to test for compatibility. Haemolysis was excluded by centrifugating a 1-ml blood sample at 5,000 *g *for 10 minutes (Heraeus Labofuge 300; Thermo Electron Corporation, Langenselbold, Germany).

### Goals of haemostasis and haemodynamics [see Additional file 1]

Animals remained under general anaesthesia, receiving pressure-controlled ventilation until conclusion of the study protocol (15-30 breaths/minute, tidal volume 6-12 ml/kg, and FiO_2 _0.3-1.0, depending on oxygenation). Monitoring throughout the experiment included ECG, arterial, central venous and intracranial pressure, oxygen saturation, and core body temperature. Urinary output, haemoglobin, hematocrit and lactate, serum electrolytes, acid-base balance, blood gases, and blood glucose levels were monitored hourly and immediately corrected as required. PT, INR, and serum albumin were measured before, after, and every 8 hr after hepatectomy until death. All blood samples were obtained from the arterial catheter. Norepinephrine, in combination with fresh-frozen plasma, hydroxyethyl starch 6% (Voluven HES 130/0.4; Fresenius, Bad Homburg, Germany), and sodium chloride solution 0.9% were used to ensure haemodynamic stability. Blood glucose levels were maintained at >100 mg/dl with glucose 20% solution. Packed erythrocyte units were given if haemoglobin levels were <6 g/dl. If renal failure occurred, pigs received furosemide (maximum 1,000 mg/d) to maintain diuresis as long as possible. Antibiotic prophylaxis (2 g ceftriaxon; Hoffmann-La Roche, Basel, Switzerland) was given daily. Death was defined as decline of mean arterial pressure below 30 mmHg under maximal ICU therapy.

Postmortem examinations were performed to verify the patency of the vascular graft, absence of bleeding complications, and amount and type of ascites. Histological studies of the kidney and brain were performed in exemplary cases.

### Statistical analysis

Mean values of the selected variables determined before, during, and after hepatectomy were compared by *t*-test (JMP 4.0; SAS Institute, Cary, NC, USA). A *P *value <0.01 was considered significant. Results are reported as means ± standard deviations. Figures are given as means ± standard error of the mean.

## Results

Postoperative survival was 100% after 24 hr. No signs of portal hypertension or intestinal congestion were noticed during the entire observation period.

**Figure 1:**
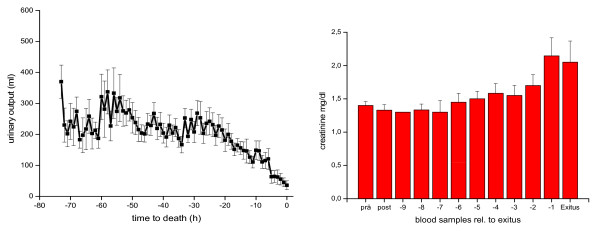
**Urinary output (ml) and creatinine (mg/dl) in blood samples in relation to time to death**.

All animals died of progressive liver failure between 44 and 73 hr after hepatectomy; mean survival time was 58 ± 4 hours. Approximately 24 hours after hepatectomy, all animals began to develop multiple-organ failure due to liver insufficiency with continuous deterioration of renal and pulmonary function. Urinary output remained stable with furosemide, but finally decreased continuously when renal failure occurred at the end of the experiment and was paralleled by an increase in serum creatinine levels (Figure 1).

**Figure 2:**
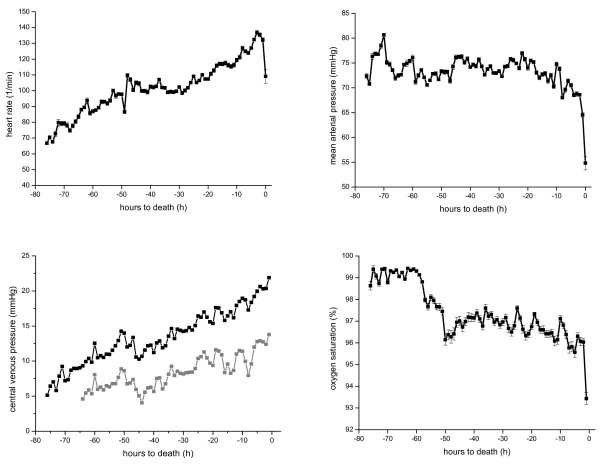
**Haemodynamic parameters HR, MAP, CVP (light gray line: CVP-PEEP), and SpO_2 _in relation to hours to death**.

Haemodynamic variables such as heart rate (HR), mean arterial pressure (MAP), central venous pressure (CVP), and oxygen saturation (SpO_2_) remained stable for the majority of the experiment in all animals (Figure 2) but then deteriorated a few hours before death [see Additional file 2].

**Figure 3:**
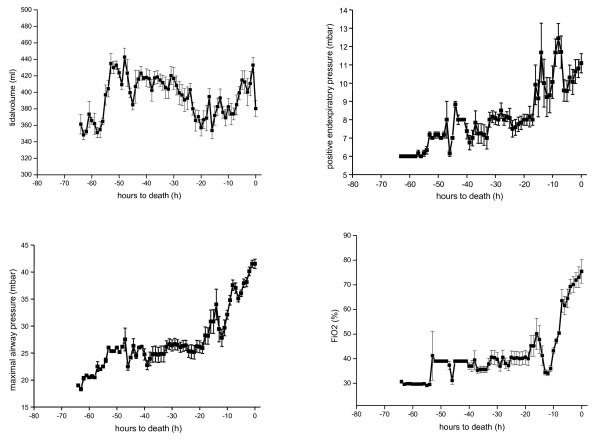
**Ventilation variables tidal volume, PEEP, P_max_, and FiO_2 _in relation to hours to death**.

**Figure 4:**
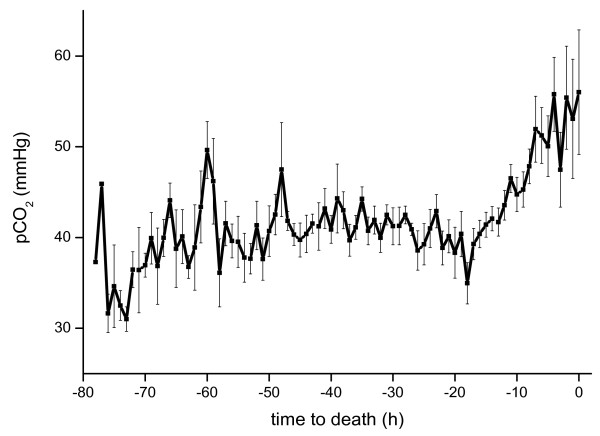
**Escalating pCO_2 _(mmHg) in relation to hours to death**.

To maintain sufficient oxygenation and ventilation, tidal volume was adjusted to 8-12 ml/kg by increasing positive end-expiratory pressure (PEEP), maximal airway pressure (P_max_) and FiO_2 _(Figure 3). Rising arterial CO_2 _tension suggested increasing dead space, particularly as minute ventilation was increased to address hypercapnia or indicated increased shunt volume (Figure 4, [see Additional file 3]).

Intracranial pressure was recorded in exemplary animals. It started at elevated levels of 15-17 mmHg due to the unphysiological supine position and then increased to 41-43 mmHg upon death.

**Figure 5:**
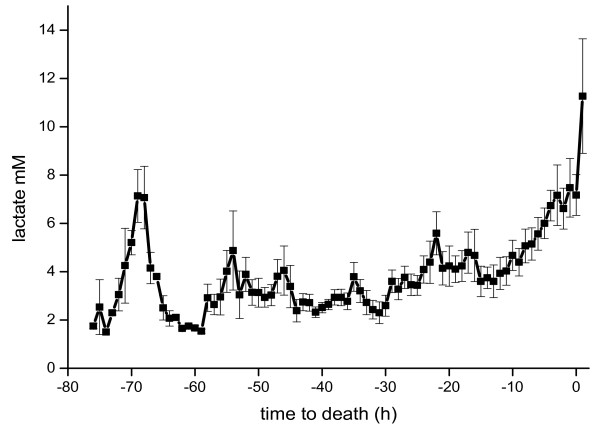
**Lactate (mM) values relative to hours to death**.

Lactate levels of 8.1 ± 2 mM in the early postoperative period decreased after haemodynamic stabilization and increased to 11.2 ± 4 mM upon death (Figure 5). PT and plasma proteins remained stable thanks to continuous replacement with fresh frozen plasma.

Upon autopsy, massive ascites (2,000 to 3,000 ml) were found in all animals; no signs of intestinal congestion were noticed, and all interposition grafts were found to be regularly patent without evidence of bleeding. No pericardial effusion or macroscopic signs of myocardial damage inducing decreased contractility could be observed. Kidneys were swollen and showed hemorrhagic infarctions; histological examinations confirmed tubular necrosis. Histological examination of the brain revealed massive oedema.

## Discussion

Several different animal models have been developed to evaluate the efficacy and safety of artificial or bioartificial liver support systems during acute liver failure. Acute hepatic failure is achieved through intoxication, ischemia, extensive liver resection, or hepatectomy. Terblanche *et al*. [26] first postulated high reproducibility of liver failure models, death due to liver failure, and defined the development time for the detection of therapeutic effects. Large animal models of acute hepatic failure using hepatotoxins such as galactosamine have a poor clinical relevance. Acetaminophen intoxication models suffer from difficulties in adjusting the adequate dosage of toxins, with undesirable effects of toxins provoking early death due to cardiocirculatory failure within 6.5 hr [27], which possibly could be avoided by adequate intensive care therapy. Methemoglobin formation and resulting respiratory failure were identified as major problems in intravenously administered acetaminophen intoxication models. In contrast to our model, standardized intensive care management, including fluid resuscitation, catecholamines, and mechanical ventilation to minimize toxic effects of acetaminophen, has never been established. It is likely that survival in acute liver failure could be increased by using adequate intensive care therapy.

While only a model of total hepatectomy with a loss of the entire functional liver tissue may be able to isolate the effectiveness of a liver support system, anhepatic models have been criticized because of the lack of toxic compounds coming from the necrotic liver [26] and the absence of chance for spontaneous regeneration. Anhepatic situations in patients caused by major surgical traumas or hyperacute rejection after liver transplantation are very rare in clinical practice but are discussed controversially as a rescue option.

Our newly developed technique [23] allows total hepatectomy in haemodynamically stable animals due to lateral clamping of large blood vessels. Standardized critical care management has made a significant impact on outcome. Considering this fact, it is astonishing that none of the previous models of acute hepatic failure gave pigs any standardized critical care therapy; such therapy has been established in humans for the past decade [25].

A review of the management of experimental anhepatic coma in various pig models showed treatment strategies to vary completely from no treatment at all [24] to mechanical ventilation and supportive therapy with colloid fluid resuscitation and catecholamines [19]. After surgery, our animals remained under general anaesthesia and received maximal critical care support, including mechanical ventilation, catecholamines, diuretics, and other medication if required.

Since encephalopathy could not be judged clinically by character of respiration, in contrast to previous studies in which pigs were allowed to wake up and breathe spontaneously after hepatectomy [14,21,24,28], in our study intracranial pressure was monitored to obtain at least a surrogate for cerebral function.

Survival time without mechanical ventilation in other models ranged from about 10 [28] to 17 hr [14] in previous studies in contrast to about 34 [29], 46 [19], and our result of 58 ± 4 hr with continuous mechanical ventilation. These results clearly show that continuous mechanical ventilation is needed in anhepatic models because increasing intracranial pressure otherwise results in hypoxia and subsequent death.

End-stage liver failure is characterized by a loss of vascular autoregulation, resulting in vasodilatation and severe hypotension. In most porcine models, hypotension was treated only with crystalloid or colloid fluid resuscitation, without catecholamines to compensate for developing circulation failure. Using phenylephrine or norepinephrine in combination with colloid fluid resuscitation, fresh frozen plasma and erythrocyte units provided considerably longer survival, at least in our study as compared with all previously reported studies. Our results clearly show that standardized intensive care medicine in our model of acute hepatic failure prolonged anhepatic survival and improved outcome significantly.

While these differences in intensive care management in animal models may be simply epiphenomena of the device and/or strategy being studied, they must be acknowledged as such as well. For example, if suboptimal critical care support produces low survival times that may be prolonged with a given liver support system, a false-positive result favouring a given liver support system may result. When extrapolating such observations, it is possible that laboratory results are transferred to a clinical intensive care medicine setting where a liver support system is unable to provide any benefit. Accordingly, when these bench-to-bedside differences are not appropriately accounted for, additional technology may no longer be superior to standard therapy. Since our survival times are about two to four times longer than those for currently established models, we suggest that our model might serve as a tough tool for evaluating liver support systems. Therefore, prolonged survival gained by different bioreactors has to be reevaluated and compared with a standard intensive care control group. A combination of artificial or bioartificial liver support systems with standard intensive care therapy should improve considerably the survival times of our animal model. The point of principle if a liver support system is superior to intensive care in the stabilisation of the haemodynamic situation, respiration, or appearance of brain oedema in the further course of anhepathy can easily be answered.

## Conclusions

Careful intensive care support enabled survival for about 60 hr in an anhepatic porcine model and may thus be a valuable tool for screening the efficacy and safety of liver support technologies.

## Key messages

• Standardized intensive care treatment improves survival in a large animal model for acute liver failure.

• This treatment alone is superior to most developed treatment strategies using liver support devices.

• Using standardized intensive care treatment prolongs the therapeutic window for the application of new liver support devices or other strategies.

## Abbreviations

ICU: intensive care unit; ICP: intracranial pressure; PT: prothrombin time; INR: international normalised ratio; FiO_2_: oxygen concentration; PEEP: positive end-expiratory pressure; HR: heart rate; MAP: mean arterial pressure; CVP: central venous pressure; SpO_2_: oxygen saturation; P_max_: maximal airway pressure; pCO_2_: partial pressure carbon dioxide.

## Competing interests

The authors declare that they have no competing interests.

## Authors' contributions

CT conceived of the study and coordinated the study group and as a surgeon he operated the pigs. KT participated in the design if the study and coordination and helped to draft the manuscript and as a surgeon she operated the pigs. AE as an anaesthesiologist carried out the intensive care therapy. TS was involved in the neurological measurement of the pigs. MM as a neurosurgeon participated in the design of the study concerning neurological aspects and placed the cranial probes. AK helped to draft the manuscript. MS designed the study and performed the statistical analysis. All authors read and approved the final manuscript.

## Supplementary Material

Additional file 1 **ICU Management**. Algorithms for volume resuscitation, vasopressor support and management of mechanical ventilation. Click here for file

Additional file 2 **Course of haemodynamic parameters and electrolytes**. Haemodynamic parameters, electrolytes and body temperature with respect to time and resuscitation. Click here for file

Additional file 3 **Course of ventilation parameters**. Ventilation parameters and body temperature with respect to time and resuscitation. Click here for file
