# Supplementary material for: Standardized intensive care unit management in an anhepatic pig model: new standards for analyzing liver support systems
Source: Crit Care. 2010 Jul 22;14(4):R138. doi: 10.1186/cc9196 (PMC2945114; doi:10.1186/cc9196)
Supplement: Additional file 1 — ICU Management. Algorithms for volume resuscitation, vasopressor support and management of mechanical ventilation. [file cc9196-S1.DOC]

**Additional file 1**

*Cardiovascular System*

MAP > 70 mmHg to maintain adequate perfusion of all organ systems

CVP 6-12 mmHg as a parameter for preload

*Fluid management*

After total hepatectomy the MAP should be kept > 70 mmHg, on the run up to and following liver failure the following protocol should be followed when the MAP starts to drop.

- Give a bolus of 250 mL hydroxyethylstarch 6% until an increase in CVP of 3 mmHg is evident.
- Re-evaluate 30-60 minutes. If this increase is maintained for more than 10 minutes then consider the vascular system to be adequately filled. Pulmonary edema is unlikely whilst there is no cardiac failure.

*Substitution of erythrocyte- and fresh frozen plasma units*

- Give 1 erythrocyte unit (300 mL) if hemoglobin level tend to decrease < 6 g/dL
- Give 8 fresh-frozen plasma units (300 mL) within 24 hours

*Use of catecholamines*

Gradually increase norepinephrine when CVP increases by 3 mmHg with a 250 mL fluid challenge by little or no accompanying increase in MAP. Do not increase norepinephrine if the last incremental increase had no effect on the MAP or MAP is < 30 mmHg.

*Electrolytes and glucose*

Ca^2+^ 1.0 - 1.3 mM using neat infusion of CaCl_2_ on demand

K^+^ 3.5 - 4.5 mM using neat infusion of KCl on demand

Glucose > 100 mg/dL by continuous infusion of glucose 20% solution

*Acid base balance*

pH 7.35 - 7.45, base excess (BE) +3 - -3.

- Correct respiratory acidosis when pCO2 > 45 mmHg
- If HCO3^-^ is less than 19 mM/L give 100 mL NaHCO3 4.2%, repeat arterial blood gas analysis to confirm therapy

*Respiratory System*

pO2 > 90 mmHg to maintain sufficient oxygenation

pCO2 35-45 mmHg to avoid respiratory acidosis or alkalosis

SpO2 > 96%

- The ventilation mode will be pressure controlled ventilation. Adjust the maximal airway to maintain an expiratory tidal volume of approximately 6-12 mL/kg. Adjust respiratory rate to increase or decrease the minute volume to keep arterial pCO2 within the prescribed limits.
- Adjust FiO2 to maintain pO2 > 90 mmHg up to 0.6. Increase PEEP as necessary step by step (maximal PEEP 14 mbar) before escalating the FiO2 > 0.6.
