# Supplementary material for: Standardized intensive care unit management in an anhepatic pig model: new standards for analyzing liver support systems
Source: Crit Care. 2010 Jul 22;14(4):R138. doi: 10.1186/cc9196 (PMC2945114; doi:10.1186/cc9196)
Supplement: Additional file 2 — Course of haemodynamic parameters and electrolytes. Haemodynamic parameters, electrolytes and body temperature with respect to time and resuscitation. [file cc9196-S2.DOC]

**Additional file 2**

| Time to Exitus (h) | Total Volume infused (ml) | | | Body Temp  (°C) | Ca^2+^  (mM) | K^+^  (mM) | pH | HB  (g/dL) | HR  (min^-1^) | MAP (mmHg) | CVP-PEEP  (mmHg) | PT  (%) |
| --- | --- | --- | --- | --- | --- | --- | --- | --- | --- | --- | --- | --- |
|  | (Crystall. and colloid. sol.) | (Erythrocyte concentrate) | (Fresh Frozen Plasma) |  |  |  |  |  |  |  |  |  |
| -64 | 2750±354 | 150±212 | 600±424 | 36.5±0.4 | 1.4±0.1 | 3.9±0.3 | 7.41±0.05 | 10.5±0.6 | 85±18 | 74±11 | 5±3 | 100±1 |
| -56 | 2500±707 | 150±212 | 900±0 | 36.6±0.9 | 1.3±0.1 | 3.8±0.5 | 7.40±0.06 | 9.6±0.6 | 93±16 | 74±14 | 6±3 | 82±15 |
| -48 | 2500±707 | 0±0 | 900±0 | 36.9+0.8 | 1.3±0.2 | 3.6±0.5 | 7.40±0.06 | 10.1±1.8 | 101±23 | 75±12 | 7±5 | 68±23 |
| -40 | 1900±894 | 180±268 | 480±268 | 37.5±0.7 | 1.3±0.1 | 3.6±0.3 | 7.43±0.06 | 11.0±1.8 | 102±21 | 77±12 | 6±4 | 59±23 |
| -32 | 2000±935 | 180±268 | 720±342 | 37.8±0.7 | 1.3±0.1 | 3.6±0.3 | 7.44±0.05 | 10.2±1.3 | 100±23 | 74±13 | 9±4 | 69±33 |
| -24 | 1900±224 | 180±164 | 840±251 | 37.9±0.7 | 1.3±0.2 | 3.5±0.3 | 7.43±0.08 | 9.8±1.4 | 106±21 | 76±12 | 11±4 | 51±10 |
| -16 | 1600±548 | 180±268 | 780±342 | 37.9±0.6 | 1.3±0.1 | 3.7±0.4 | 7.43±0.06 | 9.7±1.2 | 115±28 | 75±15 | 11±3 | 45±9 |
| -8 | 2100±742 | 540±329 | 660±251 | 38.0±1.0 | 1.3±0.1 | 3.8±0.3 | 7.36±0.14 | 9.3±1.6 | 123±25 | 72±15 | 11±3 | 40±17 |
| 0 | 1600±822 | 240±251 | 600±367 | 37.8±1.3 | 1.2±0.2 | 4.1±1.1 | 7.29±0.20 | 7.6±2.0 | 133±27 | 68±17 | 11±4 | 34±23 |

All values are expressed as mean±SD over the observation period of 8 hours from exitus.
