# Supplementary material for: Standardized intensive care unit management in an anhepatic pig model: new standards for analyzing liver support systems
Source: Crit Care. 2010 Jul 22;14(4):R138. doi: 10.1186/cc9196 (PMC2945114; doi:10.1186/cc9196)
Supplement: Additional file 3 — Course of ventilation parameters. Ventilation parameters and body temperature with respect to time and resuscitation. [file cc9196-S3.DOC]

**Additional file 3**

| Time to Exitus (h) | Total Volume infused (ml) | | | Body Temp (°C) | FiO_2_ (%) | Pmax (mbar) | Peep (mbar) | MVexp (l) | pO_2_ (mmHg) | pCO_2_ (mmHg) |
| --- | --- | --- | --- | --- | --- | --- | --- | --- | --- | --- |
|  | (Crystall. and colloid. sol.) | (Erythrocyte concentrate) | (Fresh Frozen Plasma) |  |  |  |  |  |  |  |
| -64 | 2750±354 | 150±212 | 600±424 | 36.5±0.4 | 0.29±0.07 | 19.7±1.2 | 5.8±1.3 | 4.5±0.6 | 132.3±29.1 | 40.6±5.7 |
| -56 | 2500±707 | 150±212 | 900±0 | 36.6±0.9 | 0.31±0.09 | 22.3±2.0 | 6.2±0.5 | 5.2±0.6 | 133.5±45.7 | 40.9±8.1 |
| -48 | 2500±707 | 0±0 | 900±0 | 36.9+0.8 | 0.38±0.03 | 25.3±2.3 | 7.1±1.0 | 5.5±0.5 | 137.5±35.5 | 41.5±7.0 |
| -40 | 1900±894 | 180±268 | 480±268 | 37.5±0.7 | 0.38±0.07 | 24.6±3.4 | 7.6±1.4 | 6.1±0.8 | 112.9±32.9 | 42.0±5.4 |
| -32 | 2000±935 | 180±268 | 720±342 | 37.8±0.7 | 0.38±0.08 | 25.6±4.3 | 7.6±1.6 | 6.2±1.4 | 94.6±24.6 | 41.6±4.8 |
| -24 | 1900±224 | 180±164 | 840±251 | 37.9±0.7 | 0.40±0.09 | 25.9±3.7 | 7.9±1.4 | 5.9±1.3 | 98.3±19.9 | 40.4±6.2 |
| -16 | 1600±548 | 180±268 | 780±342 | 37.9±0.6 | 0.43±0.14 | 29.0±7.5 | 9.1±3.7 | 6.5±1.7 | 99.7±16.6 | 40.4±6.3 |
| -8 | 2100±742 | 540±329 | 660±251 | 38.0±1.0 | 0.52±0.18 | 33.8±6.0 | 10.2±3.8 | 7.3±1.6 | 98.5±34.0 | 48.8±9.8 |
| 0 | 1600±822 | 240±251 | 600±367 | 37.8±1.3 | 0.72±0.19 | 39.8±4.6 | 10.5±3.3 | 8.1±1.8 | 104.9±67.3 | 55.2±22.7 |

All values are expressed as mean±SD over the observation period of 8 hours from exitus.
